# The effect of ocular rinse volume on surface irritation after povidone-iodine preparation for intravitreal injections: a randomized controlled trial

**DOI:** 10.1186/s40942-023-00470-z

**Published:** 2023-09-28

**Authors:** Farzad Jamshidi, Haoxing D. Jin, Andrew Bruce, Michael Kutteh, Kai Ding, Kamran M. Riaz, Ronald M. Kingsley, Vinay A. Shah

**Affiliations:** 1 Department of Ophthalmology, Dean McGee Eye Institute, University of Oklahoma Health Sciences Center, 608 Stanton L Young Blvd, Oklahoma City, OK 73104 USA; 2 Department of Ophthalmology and Visual Sciences, University of Iowa, Iowa City, IA USA; 3 University of Oklahoma College of Medicine, Oklahoma City, OK USA; 4 Hudson College of Public Health, University of Oklahoma Health Sciences Center, Oklahoma City, OK USA

**Keywords:** Intravitreal injection, Retina

## Abstract

**Purpose:**

To evaluate whether the volume of wash out rinse after povidone iodine (PI) application for intravitreal injections (IVI) affects patients’ ocular surface irritation.

**Methods:**

This was a prospective, single-masked, randomized-controlled trial consisting of 142 subjects. A total of 51, 45, and 46 patients received 3-mL, 10-mL, and 15-mL of ocular rinse respectively. Reductions in the Ocular Surface Disease Index (OSDI) and the Standardized Patient Evaluation of Eye Dryness II (SPEED II) surveys, conducted before and at 24–72 h post-injection, were analyzed.

**Results:**

There was no statistical difference in objective dry eye findings of Schirmer test (*p*-value = 0.788), tear break-up time (*p*-value = 0.403), Oxford fluorescein grade (*p*-value = 0.424) between the study groups prior to injections. Dry eye symptoms as measured by reductions in the OSDI and SPEEDII scores were not different between the study groups (p-value = 0.0690 and 0.6227, respectively).

**Conclusion:**

There is no difference in patients’ ocular surface irritation between 3-mL, 10-mL, and 15-mL post injection rinse. Given the large number of IVIs performed, modification of practice patterns based on these findings could lead to significant reduction in global cost burden for IVIs.

**Supplementary Information:**

The online version contains supplementary material available at 10.1186/s40942-023-00470-z.

## Introduction

Intravitreal injections (IVI) are the most commonly performed ophthalmic procedures in the United States [1]. 5% Povidone iodine (PI) application is one of the most critical and well established pre-IVI preparation techniques [2–4]. However, PI is toxic to the ocular surface [5, 6] and is known to lead to worsening of ocular surface disease and symptoms [7, 8].

Previous guidelines have emphasized anti-septic techniques before and analgesia during injections [4]. However, post injection ocular surface irritation secondary to PI exposure has been the subject of fewer inquiries. Ali et al. reported less ocular surface irritation with chlorhexidine use with comparable safety profiles when compared to PI [8, 9]. However, PI has been reported to have a greater bactericidal effect [9, 10] and remains the most commonly recommended anti-septic [4]. Fam et al., in a non-randomized patient reported outcome study, suggested spraying of dilute hypochlorous acid to reduce patient irritation [10, 11]. We had previously performed a randomized controlled trial on the utility of dissolvable collagen punctal plugs (CPP), and found that while improving symptoms in patients with moderate-to-severe dry eye disease, CPPs do not alleviate post injection irritation in patients with mild or no dry eye symptoms [11, 12].

Interestingly, while rinsing with a buffered solution after IVI is common practice [13], to our knowledge there are neither standardized recommendations on the rinse volume nor any studies that have evaluated whether rinsing volume affected patient comfort levels. Hence, we designed this study to evaluate whether variability in washout rinse volume affected patients’ subjective experiences of ocular discomfort after IVI using the Ocular Surface Disease Index© (OSDI) and Standardized Patient Evaluation of Eye Dryness II (SPEED II) questionnaires [14]. OSDI and SPEED II have similar reliability coefficients and may be used as a measure of dry eye severity in clinical practice and epidemiological studies [15, 16]. The focus of this study was patient-centered and aimed to see if the patients experienced more surface disease if lower volumes of washout were used.

## Materials, subjects, and methods

This study was a single-center, prospective, randomized-controlled, single-masked clinical trial conducted at the Dean McGee Eye Institute, Department of Ophthalmology, University of Oklahoma Health and Sciences Center, Oklahoma City, OK. USA. Ethics approval was obtained from the university’s Institutional Review Board (IRB) (IRB #9810) with adherence to the tenets of the Declaration of Helsinki. All study participants were provided with written informed consent.

Patients requiring IVI for their clinical condition during the study period (March-July 2021) were identified as potential participants. Exclusion criteria included active ocular infection, eyelid trauma, graft versus host disease, thyroid eye disease, pregnancy, and lack of the ability or willingness to participate in the study, which included answering the post-injection telephone questionnaire 24–72 h after IVI.

**Fig. 1:**
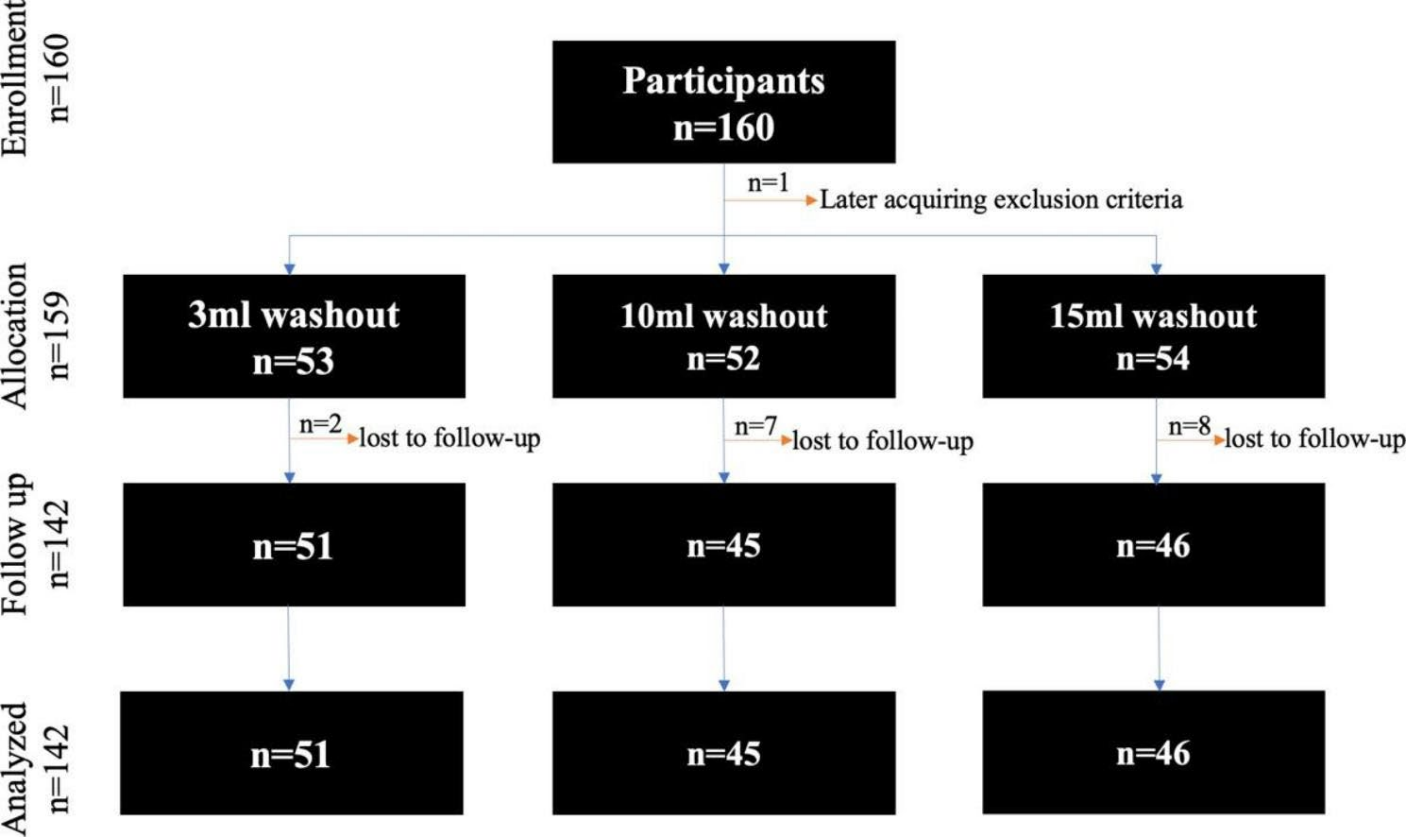
Enrollment, allocation, follow up and analysis distribution of subjection in the study

Patients were masked and randomized to 3-mL, 10-mL, and 15-mL washout cohorts in a 1:1:1 ratio. A computer-based random number-generating algorithm was used for randomization. One eye per patient was evaluated in cases of bilateral injections. The cohort distribution is displayed in Fig. 1. Participants were asked to complete the OSDI and SPEED II questionnaires [14] on an institution-approved secure web platform on an Apple iPad® (Cupertino, CA) tablet prior to IVI preparations. Objective testing of the ocular surface consisted of tear break-up time (TBUT), basic secretion test (BST) with Schirmer strips and Oxford fluorescein corneal staining grade [15–17]. These were performed as described previously [12] prior to IVIs. IVI preparation was uniform across all patients and completed as described previously [12]. In short, one drop of proparacaine was applied followed by one drop of 5% PI. This was repeated 3 times within 10 min. A speculum was used to hold the eyelids open for the IVIs. IVIs were completed within 15 min of the last PI and proparacaine instillation. After the injection was performed, various volumes of ophthalmic solution eyewash (Medi-Frist®, Myers, FL) was used to thoroughly rinse the ocular surface with patients in the primary, up, down, left, and right gaze directions. Post-injection OSDI and SPEED II questionnaires were conducted via phone call between 24 and 72 h after IVIs.

Power analysis: Our preliminary data showed that the mean (SD) OSDI score reduction is 1.6 (22.1), 7.7 (12.7), and 13.8 (15.6) for the 3 ml, 10 ml, and 15 ml treatment group, respectively. To detect the difference between the 3 ml and the 15 ml group with the observed SDs and 0.05 alpha level (two-sided), 40 patients per group were needed to achieve 80% power based on a two-sample t-test with unequal variance. Our preliminary data also showed that the mean (SD) SPEED II score reduction is 1.8 (4.4), 1.4 (6.6), and 5.1 (3.9) for the 3 ml, 10 ml, and 15 ml treatment group, respectively. With 40 patients per group, we had 94% power to detect the difference in SPEED II score reduction between the 3 ml and the 15 ml group, again based on a two-sample t-test with unequal variance with 0.05 alpha level (two-sided). To account for 20% drop-out rate, an enrollment of 50 patients per group was planned. The PASS 16 software was used for the power analyses.

Statistical analysis: Descriptive statistics were used to summarize basic demographic characteristics. Continuous data were summarized using mean and standard deviation. Comparisons between treatment groups were performed using the Chi-square test for categorical variables and analysis of variance (ANOVA) for continuous variables. Multiple comparisons were adjusted by Tukey’s method. The OSDI score was calculated via the formula: OSDI = [(sum of scores for all questions answered) × 100] / [(total number of questions answered) × 4] [18]. The total Speed Score was calculated as the sum of the responses to the 8 SPEED questions (Frequency + Severity). Multivariate analyses were conducted using linear regression, where the following baseline variables were considered: race, gender, study eye, fluorescein staining, TBUT, Schirmer test, OSDI and SPEED II scores. Analyses were performed in SAS version 9.4 (SAS Institute, Inc., Cary, NC). All *p*-values were considered statistically significant when they were < 0.05.

## Results

160 patients enrolled in the study. One patient subsequently met the exclusion criteria and was not randomized. 17 dropped out of the study giving a total of 142 patients for the follow-up and for the final analysis (Fig. 1). These included 51 patients in the 3-mL, 45 in the 10-mL, and 46 in the 15-mL washout groups. No endophthalmitis or any other complications associated with the IVIs occurred during the study.

Demographic data are shown in Table 1. 80.3% of study patients were Caucasian and 51.4% of participants were female. 24% of patients had Oxford Grade III or worse on fluorescein staining, 47.9% had TBUT < 5 s, and 10.6% had BST tests at < 5 mm, suggesting an appreciable presence of patients with dry eye disease. Of note, there was no significant difference among cohorts with regards to race, gender, study eye, fluorescein staining, TBUT, and BST with regards to OSDI and SPEEDII prior to receiving IVIs (Table 1).

**Table 1:** Baseline characteristics, objective and subjective assessments prior to injections

Variable	Description	All	3ML	10ML (n = 45)	15ML (n = 46)	p-value
		(n = 142)	(n = 51)			
RACE:	African American	9 (6.3%)	1 (2.0%)	2 (4.4%)	6 (13.0%)	**0.0893**
	Mixed	3 (2.1%)		1 (2.2%)	2 (4.3%)	
	Native American	10 (7.0%)	2 (3.9%)	2 (4.4%)	6 (13.0%)	
	Native Hawaiian/	1 (0.7%)			1 (2.2%)	
	Pacific Islander					
	Other	5 (3.5%)	2 (3.9%)	2 (4.4%)	1 (2.2%)	
	White/Caucasian	114 (80.3%)	46 (90.2%)	38 (84.4%)	30 (65.2%)	
GENDER:	Female	73 (51.4%)	22 (43.1%)	26 (57.8%)	25 (54.3%)	**0.3298**
	Male	69 (48.6%)	29 (56.9%)	19 (42.2%)	21 (45.7%)	
STUDY EYE:	OD	83 (58.5%)	30 (58.8%)	26 (57.8%)	27 (58.7%)	**1**
	OS	59 (41.5%)	21 (41.2%)	19 (42.2%)	19 (41.3%)	
FLUORESCEIN STAINING:	0	33 (23.2%)	13 (25.5%)	8 (17.8%)	12 (26.1%)	**0.4235**
	I	45 (31.7%)	15 (29.4%)	15 (33.3%)	15 (32.6%)	
	II	30 (21.1%)	10 (19.6%)	14 (31.1%)	6 (13.0%)	
	III	18 (12.7%)	7 (13.7%)	6 (13.3%)	5 (10.9%)	
	IV	16 (11.3%)	6 (11.8%)	2 (4.4%)	8 (17.4%)	
TBUT	0–5 s	68 (47.9%)	25 (49.0%)	22 (48.9%)	21 (45.7%)	**0.4032**
	6–10 s	47 (33.1%)	18 (35.3%)	17 (37.8%)	12 (26.1%)	
	> 10 s	27 (19.0%)	8 (15.7%)	6 (13.3%)	13 (28.3%)	
SCHIRMER TEST	< 5 mm	15 (10.6%)	5 (9.8%)	6 (13.3%)	4 (8.7%)	**0.7883**
	>= 5 mm	127 (89.4%)	46 (90.2%)	39 (86.7%)	42 (91.3%)	
OSDI	mean and std	18.1 (17.9)	20.3 (20.0)	17.0 (15.3)	16.7 (18.0)	**0.5546**
	median and IQR	11.4 (4.5, 27.5)	15.9 (2.8, 31.8)	10.4 (5.0, 27.3)	9.1 (4.5, 22.7)	
	min and max	(0.0, 94.4)	(0.0, 94.4)	(0.0, 52.5)	(0.0, 72.7)	
	#(%) missing	1 (0.7%)			1 (2.2%)	
SPEED II	mean and std	4.1 (4.5)	3.4 (3.9)	4.6 (5.0)	4.5 (4.6)	**0.318**
	median and IQR	3.0 (0.0, 6.0)	2.0 (0.0, 6.0)	3.0 (1.0, 7.0)	4.0 (0.0, 7.0)	
	min and max	(0.0, 22.0)	(0.0, 17.0)	(0.0, 22.0)	(0.0, 20.0)	
	#(%) missing	1 (0.7%)			1 (2.2%)	

**Fig. 2:**
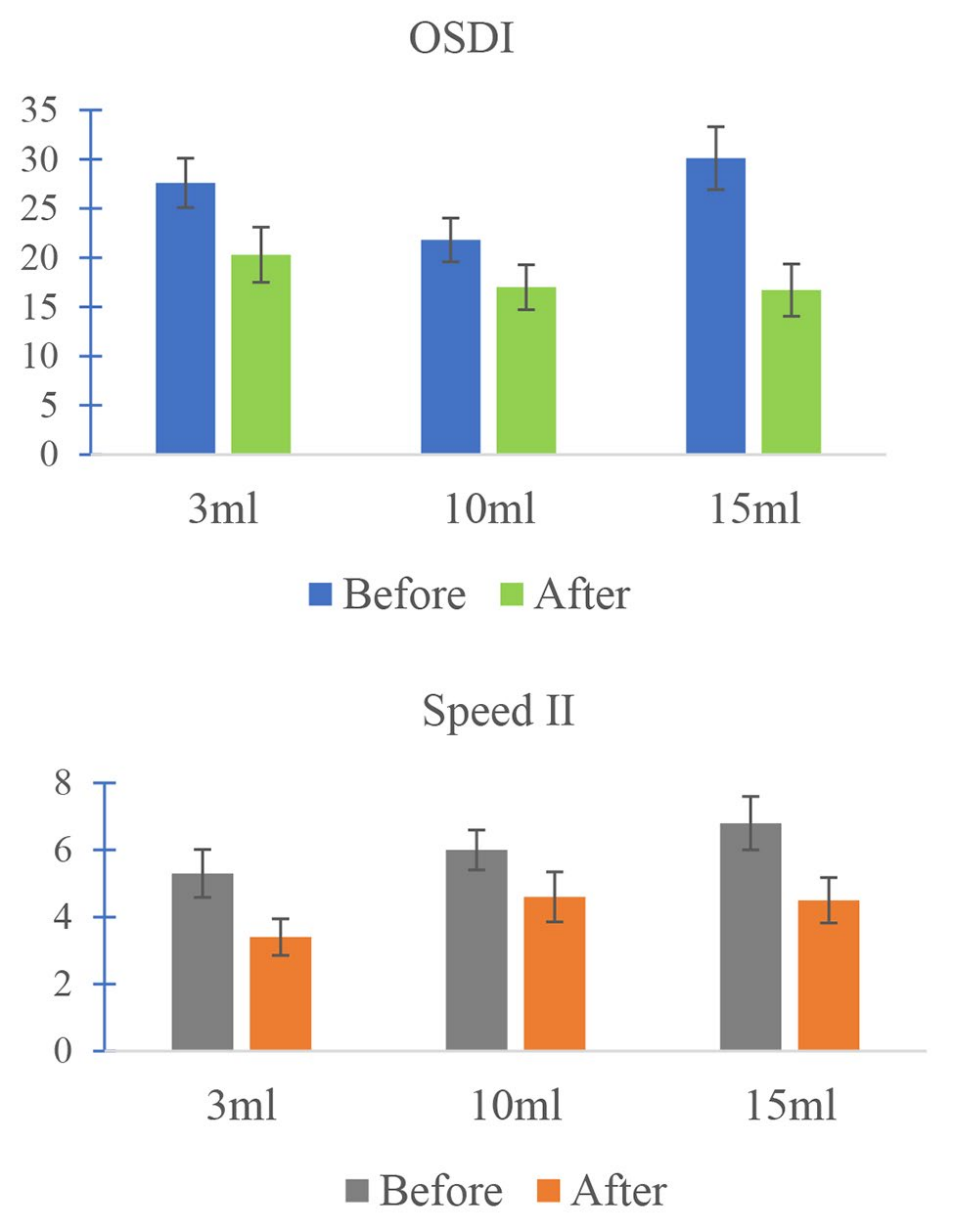
OSDI and Speed scores before and after washout in each cohort. Error bars represent standard errors of mean

There was a trend towards reduction in OSDI and SPEEDII scores after washout with various volumes (Fig. 2). However, there was no statistically significant difference between the different washout volume cohorts in the mean reduction for the OSDI (*p*-value = 0.069) or the SPEEDII score (*p*-value = 0.6227) (Table 2).

**Table 2:** Reduction in OSDI and SPEEDII scores (post vs. pre-injection) with comparison between cohorts

Variable name	Description	All	15ML	10ML	3ML (n = 51)	p-value
		(n = 142)	(n = 46)	(n = 45)		
REDUCTION IN OSDI	mean and	8.3	13.1	4.8	7.3	**0.0690**
	std	-17.5	-17.1	-15	-19.4	
	median and	7.6	9	4.2	8.7	
	IQR	(0.0, 16.7)	(2.3, 22.7)	(-2.3, 12.1)	(0.0, 15.9)	
	min and max	(-65.3, 56.9)	(-17.5, 56.9)	(-43.3, 36.4)	(-65.3, 50.8)	
	(%) missing	1 (0.7%)	1 (2.2%)			
REDUCTION IN SPEED II	mean and	2	2.5	1.4	1.9	**0.6227**
	std	-5.5	-5.1	-6.2	-5.2	
	median and	2	1	1	2	
	IQR	(-1.0, 5.0)	(0.0, 6.0)	(-1.0, 3.0)	(-2.0, 4.0)	
	min and max	(-19.0, 19.0)	(-7.0, 19.0)	(-19.0, 15.0)	(-10.0, 19.0)	
	(%) missing	1 (0.7%)	1 (2.2%)			

## Discussion

Ocular surface irritation with PI preparation is a common complaint among patients after IVIs [7, 8]. A variety of strategies have been proposed, including alternate chemoprophylaxis techniques and procedures to enhance ocular surface lubrication. [11, 12] We previously showed that use of dissolvable CPPs improves symptoms of patients with pre-existing dry eye disease, however, in patients without dry eye disease, CPPs do not improve ocular surface irritation after IVIs [12]. Washing with a buffered eyewash solution after IVIs is a common yet unstandardized step of IVIs [13]. It may seem intuitive that larger volumes of ocular rinse may lead to improvement in symptoms secondary to a presumed better removal of residual PI. However, this has not been assessed previously. To our knowledge, this is the first randomized controlled study assessing the effect of ocular rinse volume on ocular surface irritation after IVIs.

We did not find a difference between smaller (3-mL) vs. larger washout (10 or 15-mL) volumes using two different standardized ocular surface irritation questionnaires (OSDI and SPEEDII). It is possible that 3-mL of rinse with purified water solution may be sufficient to remove all residual PI or that the difference in residual PI in 3-mL vs. 10 or 15-mL of washout is not symptomatically significant. However, there was a trend of overall reduction in surface irritation symptoms after washouts in all 3 cohorts (Fig. 2). This suggests that some amount of washout may be needed, and the technique of careful rinsing in different gaze directions is probably more important than the total volume of eye rinse. However, whether smaller than 3-mL of rinse volume or no washout would produce similar results is yet to be determined. Given recommended guidelines [4, 13] and standard practice patterns, it is challenging to recruit patients into studies without any ocular rinse.

Additionally, we repeated the questionnaires within 72 h of IVIs. Both OSDI and SPEEDII assess symptoms within the past several days and hence, theoretically, differences in symptoms from the time of injection to the time of the phone survey should have been captured. However, recall bias towards symptoms at the time of the phone survey may overshadow discomfort immediately after IVIs. Future studies with surveys conducted at a shorter time interval, e.g. less than 24 h, may elucidate a difference between various washout volumes. However, our study shows that by 1–3 days after IVI, patients do not report a significant difference in surface irritation symptoms. A confounding factor could be number of injections previously received by patients. Almost all of our patients had received multiple prior injections; however, we did not analyze whether individual cohorts had a statistically significant difference in number of prior injections. We did show that the cohorts did not have significant difference in race, gender, and pre-existing dry eye disease or symptoms.

While on an individual scale, the difference of 12-mL of rinse volume may not seem significant, globally it has a significant cost and time burden. Ocular rinses are inexpensive and cost is approximately 3 cents per mL (Medi-Frist® purified water eyewash, Myers, FL). However, by 2016, 5.9 million IVIs were being performed in the USA and this number continues to increase exponentially [19]. This would translate into a cost saving of at least $2.12 million simply with the use of less ocular rinse volume.

The strength of this study includes a randomized controlled design with variation of a single variable, the rinse volume. This, together with the single masked design, eliminates ascertainment biases. Additionally, the patient’s lack of knowledge of which treatment they received reduces the possibility of placebo effects when completing questionnaires. The number of participants were sufficient to ensure lack of statistical significance in unwanted variables such as gender, eye of study, and pre-treatment objective dry eye findings. Limitations of the study include lack of post-treatment objective measures as well as single time-point evaluation after the washout. It is possible, that objective findings, while not the goals of the study, can be affected by rinse out volumes. Furthermore, we did not stratify patients into ocular surface disease severity (e.g., mild, moderate, and severe) subgroups within the three study groups. We attempted to mitigate this potential confounding factor by performing objective and subjective assessment of patients prior to IVI; we did not note any statistically significant differences between the three groups (Table 1). Future studies may wish to further assess the effects of ocular surface rinse volumes among mild, moderate, and severe dry eye syndrome patients. Additionally, while we chose three objective tests for dry eye assessment, additional testing (e.g., impression cytology, meibography, etc.) may provide superior or supplemental information to clinicians and researchers alike. Finally, earlier or later time frames could have different outcomes. Future multi-institutional studies, with additional or different objective dry eye assessment testing and patient questionnaires may be needed before standardizing the volume of ocular rinse after PI preparations and IVIs across all clinical settings.

In conclusion, we noted that different volumes of ocular rinse solution did not affect patients’ experience of ocular surface irritation after IVIs. Ultimately, the decision to use a certain volume of ocular rinse should be based on clinicians’ experience and patients’ expectations. A personalized approach with appropriate informed consent should be undertaken whenever possible. Clinicians may consider our findings as they optimize IVI protocols to improve efficiency and economy.

### Electronic supplementary material

Below is the link to the electronic supplementary material.

Supplementary Material 1

## Data Availability

The data are available at the clinical trials group of Dean McGee Eye Institute, Department of Ophthalmology, University of Oklahoma Health and Sciences Center, Oklahoma City, OK. USA.

## Declarations

### Clinical trial information

This study was registered under “*The Effect of Ocular Rinse Volume on Surface Irritation After Intravitreal Injections*” at *ClinicaTrial.gov*, retrospectively registered on November 14, 2022. The registration number is NCT05615805 and the link can be found at https://clinicaltrials.gov/ct2/show/NCT05615805.
