# Supplementary material for: The effect of ocular rinse volume on surface irritation after povidone-iodine preparation for intravitreal injections: a randomized controlled trial
Source: Int J Retina Vitreous. 2023 Sep 28;9:59. doi: 10.1186/s40942-023-00470-z (PMC10537205; doi:10.1186/s40942-023-00470-z)
Supplement: Supplementary file 1 — Supplementary Material 1 [file 40942_2023_470_MOESM1_ESM.docx]

**Response to reviewers** (Reviewer comments are in **bold script**, our responses are below each reviewer comment)

**Reviewer #1: the paper was well designed but the povidine-iodine concentration and its effects are very well known by the opthalmic community. Please state what new information this manuscript provide to justify publication.** 

We appreciate the Reviewer’s comment and agree that the side effects of povidone-iodine (PI) are well-described in the ophthalmic literature and well-known to clinicians. We briefly discussed some of these adverse effects in the introduction(1^st^ paragraph, last sentence) and provided some references (References 5-8) on this point. We note that most of these adverse effects describe objective findings concerning unwanted ocular surface side effects. as well. However, while we similarly describe patient symptoms after PI-application/intravitreal injection, the key focus of our paper is on the *subjective* patient experience with the ocular rinse. We often see patients that request a “more extensive” wash-out of PI after an injection. Therefore, we sought to specifically study whether a smaller volume balanced salt solution (BSS) wash-out would be any different than a larger volume? Interestingly, we demonstrate that the volume of the washout does not matter in an objective assessment hence 3ml seems to be enough and achieve the same goal as larger volume washouts. The ophthalmologists and ophthalmic staff therefore use a simple low volume rinse and re-assure the patients based on this data.

**Reviewer #2: 1. Please mention the problem statement clearly. It is unclear if the effort of the study was to reduce the possibility of ocular surface damage or to reduce the cost of the solution to wash off the PI. If it is is the latter, then please highlight the same with available incidence data. In most recommendations, ocular fluids for surgical use are available as large volumes and after use, the remainder is not stored beyond the same day. So, how then would this have a practical impact on the overall expenditure??**

We thank the Reviewer for this comment. We have added the question under investigation more clearly at the end of the introduction. The focus of the study was patient-centered and aimed to see if the patients experienced more surface disease if lower volumes of washout were used. It is often that we experience patients requesting higher volume rinses to ensure all of the betadine has been removed from the fornix and the conjunctiva. Surprisingly however, we found that a small volume of 3ml is just as effective as 15ml in relieving the ocular symptoms. Therefore, what this study shows to ophthalmologists and ophthalmic staff is that as little as 3ml of ocular rinse will be sufficient to address ocular surface symptoms after betadine application for injections. The primary goal is patient comfort, and secondarily time efficiency in high volume injection clinics. We have added a statement in the discussion further highlighting this point. The cost saving, also discussed in discussion, is a tertiary consideration that will have minimal impact on an individual practice setting. But given intravitreal injections are among the most commonly performed medical procedures, it will have a higher impact on a global level. The cost was an additional point of interest, while the main focus is on optimization of post-injection protocol for patient comfort and for clinic efficiency.

**2. Is it not important to rinse PI from the conjunctival sac before the injection too? If there is a delay in injection of 10-15 minutes, isn't it necessary to instill the topical anaesthetic, in many patients? Wouldn't this influence the study effects?**

Indeed, it is likely that a rinse before the procedure could help improve symptoms. However, that could increase the rates of endophthalmitis. Therefore, the only variable that we tested was washing the betadine after the injections to avoid putting the patients at any added risk. None of the patients in the study had a delay of more than 15 minutes for the in intravitreal injection after application of proparacaine. This is more clearly stated in the methods section.

**3. As admitted there was a high prevalence of dry eye before the injection. Were these patients diagnosed and on any treatment? What was the distribution in the 3 groups?**

We thank the Reviewer for this comment. As noted in the Results section, paragraph 2 provides information regarding objective findings for fluorescein staining, tear break up time, and basic secretion testing. As expected in an ophthalmology practice, some patients were previously diagnosed with ocular surface disease/dry eye syndrome and were on treatment with non-prescription treatments (e.g., warm compresses, lid hygiene, artificial tears, etc.). Patients on prescription medication for dry eye syndrome (e.g., Lifitegrast, cyclosporine, etc.) were not included in the study. For the purposes of this particular study, patients were not additionally stratified into ocular surface disease severity groups, as this was beyond the scope of the study among a population with retinal pathology. We attempted to mitigate this potential limitation by performing an objective assessment using the three tests mentioned prior to IVI. When looking at the three groups in the study (e.g., 3 mL, 10mL, 15mL groups), we did not observe any statistical differences (Table 1) regarding these objective measures before IVI injection and subsequent rinse. Similarly, even among the OSDI and SPEED scores (subjective measurements), we did not observe any statistically significant differences among the three groups. Thus, we believe we had a reasonable distribution of ocular surface disease severity among the three groups. We agree that future studies may wish to further assess the effects of BSS rinse among mild, moderate, and severe dry eye syndrome patients. We have added this information as a limitation in the Discussion. **4. Is it adequate too study ordinal scales of dry eye to access impact of PI on the ocular surface or would it be valuable to study the damage using methods like impression cytology?**

We thank the Reviewer for this comment and agree that other objective testing for dry eye (such as meibography, MMP-9 testing, impression cytology (IC), etc.) could be evaluated to better assess and stratify patients into mild/moderate/severe dry eye syndrome subgroups. However, given that this study was performed primarily among patients with retinal pathology, financial and logistical limitations precluded an extensive array of testing that is typically found in studies assessing dry eye syndrome. When designing the study, we had to select a combination of objective and subjective testing that would be economical, efficient, and efficacious to assess dry eye syndrome. Ultimately, we chose the three tests mentioned in the manuscript for objective assessment and the SPEED/OSDI questionnaires as subjective assessments. We additionally chose the latter questionnaires as we were able to perform these over the telephone within 72 hours of IVI, rather than impose additional financial/logistical burdens on patients to return for repeat objective testing. Specifically, regarding IC: this is a time-intensive testing that is not typically used in routine clinical practice and requires experts (E.g., ocular pathologist, microbiologist, etc) that are not available at every institution. Instead, we selected the three objective tests and two subjective questionnaires precisely because of they can be universally performed and interpreted by clinicians in nearly all settings and does not impose significant financial/time costs to providers and/or patients. We have added some additional language in the limitations paragraph to discuss these observations.
